# S-1 plus cisplatin with concurrent radiotherapy versus cisplatin alone with concurrent radiotherapy for stage III non-small cell lung cancer: a pilot randomized controlled trial

**DOI:** 10.1186/s13014-014-0306-3

**Published:** 2015-01-09

**Authors:** Lei Yao, Shidong Xu, Jianyu Xu, Chaoyang Yang, Junfeng Wang, Dawei Sun

**Affiliations:** Department of Chest Surgery, Third Affiliated Hospital of Harbin Medical University, Haping Road No.150, Nangang District, Harbin, Heilongjiang Province 150081 China

**Keywords:** S-1, Cisplatin, Radiotherapy, Non-small-cell lung cancer

## Abstract

**Background:**

We investigated the efficacy and safety of S-1 and cisplatin with concurrent thoracic radiation (SCCR) over cisplatin alone plus concurrent thoracic radiation (CCR) for unresectable stage III non-small-cell lung cancer (NSCLC).

**Methods:**

Between January 2009 and November 2011, 40 eligible patients with NSCLC were included and divided randomly into two groups. Twenty patients received SCCR with S-1 (orally at 40 mg/m^2^ per dose, b.i.d.) on days 1 through 14, cisplatin (60 mg/m^2^ on day 1) every 4 weeks for two cycles, and radiotherapy (60 Gy/30 fractions over 6 weeks) beginning on day 1. Twenty subjects received CCR (cisplatin and radiotherapy, the same as for SCCR).

**Results:**

The 3-year overall response rate was 59.3% and 52.4% for the SCCR and CCR groups, respectively, and the difference was statistically significant, while the median overall survival was 33 months (range, 4–41 months) and 24 months (range, 2–37 months), respectively (*P* = 0.048). The median progression-free survival was 31 months for SCCR (range, 5–39 months), whereas it was 20 months (range, 2–37 months) for CCR (*P* = 0.037). The toxicity profile was similar in both groups.

**Conclusion:**

In summary, we demonstrated that S-1 and cisplatin with concurrent thoracic radiation was more effective than cisplatin plus radiotherapy in NSCLC patients with acceptable toxicity.

**Trial registration:**

Chinese Clinical Trials Register: ChiCTR-TRC-13003997.

## Introduction

Non-small-cell lung cancer (NSCLC) accounts for 80% of all lung cancer cases, and approximately 30% of all lung cancer patients are diagnosed with stage III disease [1], for which the standard treatment is concurrent chemoradiotherapy [2]. Recent randomized phase III trials have shown that concurrent chemoradiotherapy is superior to chemotherapy followed by radiotherapy in terms of response and survival in these patients [3,4]. However, concurrent chemoradiotherapy is also associated with greater acute toxicity, *which* includes bone marrow suppression and esophagitis, than sequential chemoradiotherapy [5].

S-1 (TS-1, Taiho Pharmaceutical Co., Ltd) is a new oral fluoropyrimidine agent designed to enhance anticancer activity and to reduce gastrointestinal toxicity. It consists of tegafur (a 5-FU Pro-drug), 5-chloro-2, 4-dihydroxypyridine (an inhibitor of dihydropyrimidine dehydrogenase), and potassium oxonate (an inhibitor of phosphoribosyl transferase), in a molar ratio of 1:0.4:1. S-1 has been shown to induce a comparable response to the other single agents for metastatic NSCLC [6]. Two studies of S-1 plus cisplatin for advanced NSCLC showed a response rate of 32.7–47% and a median survival time of 11–16 months. These studies also reported only very few severe gastrointestinal or hematological toxicities [7,8].

To date, there have been no reported randomized controlled trials to assess the efficacy and safety of S-1 plus cisplatin with concurrent radiotherapy (SCCR) versus cisplatin plus concurrent thoracic radiation (CCR) for stage III NSCLC. Therefore, more rigorous studies are required to elucidate the feasibility and efficacy of S-1 for the treatment of these patients.

We conducted a single-center, randomized controlled pilot study to evaluate the feasibility and efficacy of S-1 plus cisplatin with concurrent radiotherapy for treating NSCLC patients. The results of this study will also help us to calculate the appropriate sample size for a future large clinical trial.

## Patients and methods

### Patient selection

Patient eligibility requirement for enrollment in this study was cytologically or histologically confirmed, unresectable stage IIIA or IIIB NSCLC, diagnosed between January 2009 and November 2011. The clinical or pathologic stage of the disease was determined based on the general rules for the TNM Classification of Malignant Tumors (6th edition) [9]. The other eligibility criteria were an age between 20 and 80 years, Eastern Cooperative Oncology Group performance status of 0 or 1, no previous chemotherapy or radiotherapy, and adequate hematologic, hepatic, and renal function. Patients also had to have the following standard laboratory test results: a leukocyte count of 4000–12 000/μl, a platelet count of ≥100 000/μl, a hemoglobin level of ≥9 g per 100 ml, a serum bilirubin level of ≤1.5 mg per 100 ml, serum aspartate aminotransferase and alanine aminotransferase levels of ≤100 IU/ml, an alkaline phosphatase level of no more than twice the upper limit of normal, a normal creatinine level, and a partial pressure of arterial oxygen of ≥65 torr in room air. All eligible patients underwent computed tomography (CT) scans of the thorax, including the upper abdomen, and a radioisotopic bone scan.

Patients who were pregnant or who had malignant pleural effusion, malignant pericardial effusion, a concomitant malignancy, or serious comorbidities such as clinically significant cardiac dysfunction, active infection, or neurologic or psychiatric disorders were excluded from the study. The study was approved by the institutional ethics committee of Third Affiliated Hospital of Harbin Medical University with permission number (KY2009-41).

The randomization code was generated using a computerized number generator through the stratified block randomization method of the SAS package (Version 9.1.3; SAS Institute Inc., Cary, North Carolina, USA) by a statistician with no clinical involvement in this study. After qualifying, patients were assigned to either of two treatment groups: SCCR group or CCR group. The allocation was concealed in sequentially numbered, opaque, sealed envelopes containing the randomization assignments. In addition, all the outcome assessors and data analysts were blinded in this study.

### Treatment schedule

A CT scan of the chest tumor was conducted in order to determine tumor volume before therapy. Patients in the CCR group received cisplatin (60 mg/m^2^) on day 1 and then at 4-week intervals, and they also underwent radiotherapy, which was administrated concurrently on day 1 by chest irradiation. Two different radiation target volumes were planned. The initial dose (approximately 40 Gy) was administered to the primary tumor, the ipsilateral hilum with a 2-cm margin, and the involved mediastinal lymph nodes with a 1-cm margin. Prophylactic radiation fields were not planned, except for subcarinal lymph nodes. Subsequently, a 20 Gy dose was given as a booster once a day for 5 days each week over a period of 6 weeks using a linear accelerator generating at least 4 MeV photons, in accordance with tumor shrinkage. In addition to receiving the same intervention as the CCR group, patients in the SCCR group were also administered S-1 (orally at 40 mg/m^2^ per dose, b.i.d., on days 1 through 14).

### Evaluation of response and toxicity

All eligible patients who received treatment were considered assessable for response and toxicity measures. Chest radiography, complete blood counts, and blood chemistry measurements were performed weekly during the treatment period. The response was assessed according to the Response Evaluation Criteria in Solid Tumors (RECIST) [10]. The toxicity for all patients who received any treatment was evaluated and graded according to the National Cancer Institute Common Terminology Criteria for Adverse Events, Version 3.0 [11].

### Statistical analysis

An intention to treat analysis was performed. Progression-free survival (PFS) was defined as the time from the starting date of induction chemoradiotherapy until disease progression or death. Patients whose disease had not progressed at the time of study treatment discontinuation continued to be assessed until progression was documented. Overall survival (OS) was defined as the time from the starting date of induction chemoradiotherapy until death from any cause. Sample size was calculated on the basis of an expected 15% difference between the 2 groups. OS was calculated from the day of randomization to the day of death. Data for surviving patients were censored on the date on which they were last known to be alive. PFS was computed from the date of randomization to the date of relapse, death, or completion of follow-up, whichever occurred first. Data on patients who were alive and progression free were censored at the time of the last follow-up visit. OS and PFS rates were calculated using the Kaplan–Meier method, and *P* ≤ 0.05 was considered statistically significant. All *P* values were obtained using 2-tailed t-tests.

## Results

**Figure 1:**
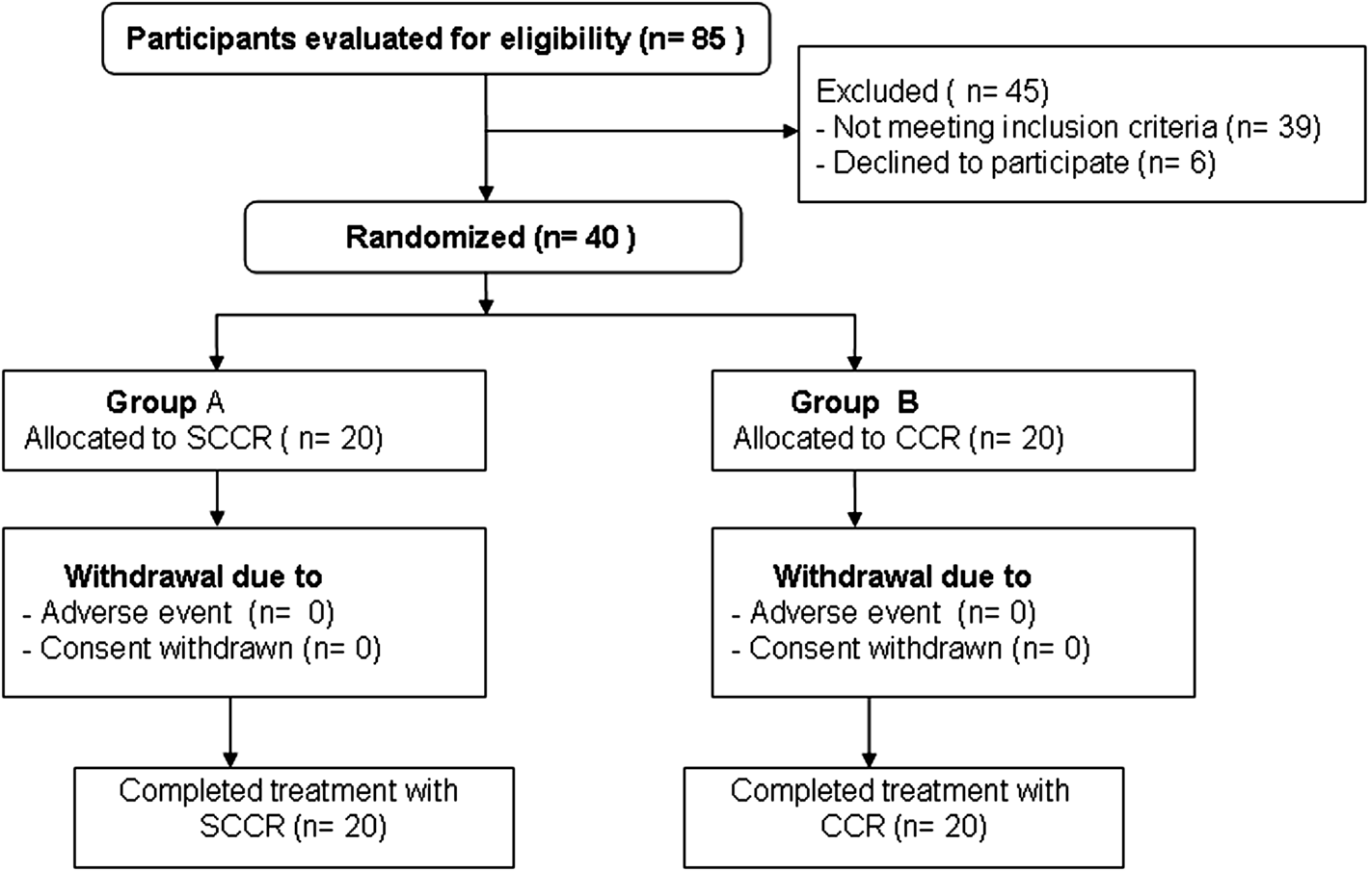
**Flow of participants through the trial.**

In this study, 85 subjects were initially screened, of whom 45 were excluded. Of these 45 patients, 39 did not meet the study criteria and 6 declined to participate. The remaining 40 patients (20 treated with SCCR, and 20 treated with CCR) were entered into the study between January 2009 and November 2011. All patients could be assessed for efficacy and safety (Figure 1).

The baseline characteristics of the patients were similar in the two treatment groups (Table 1). The mean age was 59.6 years in the SCCR group and 60.4 years in the CCR group. The performance status was 0 for 65.0% of patients treated with SCCR and 60.0% of patients treated with CCR, and it was 1 for 35.0% of patients treated with SCCR and 40.0% of patients treated with CCR. Histological examinations indicated the presence of the following cancer cell types: adenocarcinoma (45.0% in the SCCR group and 50.0% in the CCR group), squamous (35.0% in the SCCR group and 25.0% in the CCR group), and large cell carcinoma (20.0% in the SCCR group and 25.0% in the CCR group). The disease stage was IIIA (80.0% in the SCCR group and 70.0% in the CCR group) or IIIB (20.0% in the SCCR group and 30.0% in the CCR group). In total, 30 of 40 (75.0%) patients had stage IIIA disease and 10 of 40 (25.0%) patients had stage IIIB disease. The primary tumor was located in the upper lobe in 13 and 15 patients and in other lobes in 7 and 5 patients treated with SCCR and CCR, respectively.

**Table 1:** **Baseline characteristics of participants at trial entry: ITT population**

	**Variable**	**SCCR (n = 20)**	**CCR (n = 20)**	***P*** **value**
Age, yrs: mean (SD)		59.6 (19.3)	60.4 (20.1)	0.90
Race	Asian (Chinese)	20 (100.0%)	20 (100.0%)	1.00
Sex				
	Males	15 (75.0%)	14 (70.0%)	0.72
	Females	5 (25.0%)	6 (30.0%)	0.72
Performance status				
	0	13 (65.0%)	12 (60.0%)	0.74
	1	7 (35.0%)	8 (40.0%)	0.74
Histology				
	Adenocarcinoma	9 (45.0%)	10 (50.0%)	0.75
	Squamous cellcarcinoma	7 (35.0%)	5 (25.0%)	0.49
	Large cell carcinoma	4 (20.0%)	5 (25.0%)	0.71
Stage of disease				
	IIIA	16 (80.0%)	14 (70.0%)	0.47
	IIIB	4 (20.0%)	6 (30.0%)	0.47
Primary site				
	Upper lobe	13 (65.0%)	15 (75.0%)	0.49
	Middle/lower lobe	7 (35.0%)	5 (25.0%)	0.49

**Note:** ITT, intent-to-treat; SCCR, S-1, cisplatin and concurrent thoracic radiation; CCR, cisplatin and concurrent thoracic radiation; yrs, years; SD, standard deviation.

**Figure 2:**
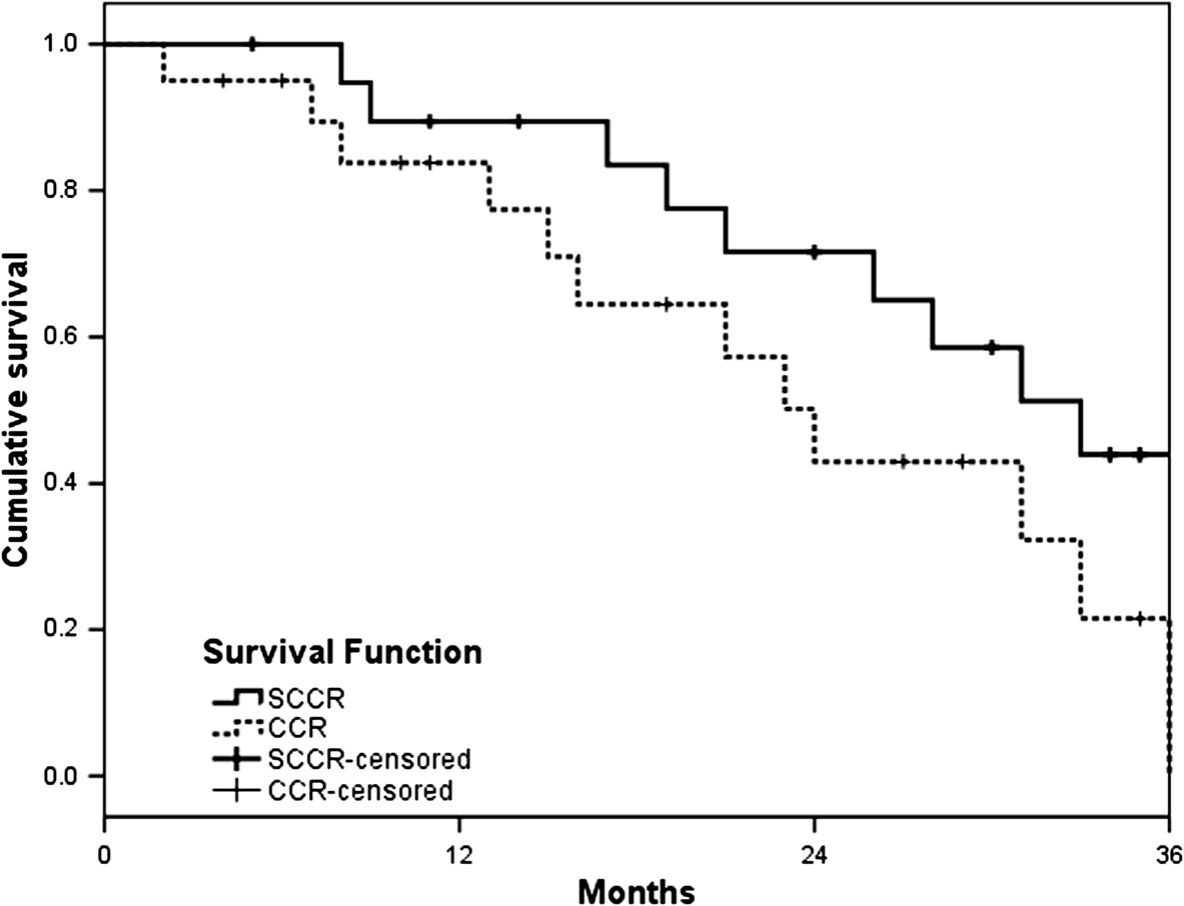
**Overall survival.**

**Figure 3:**
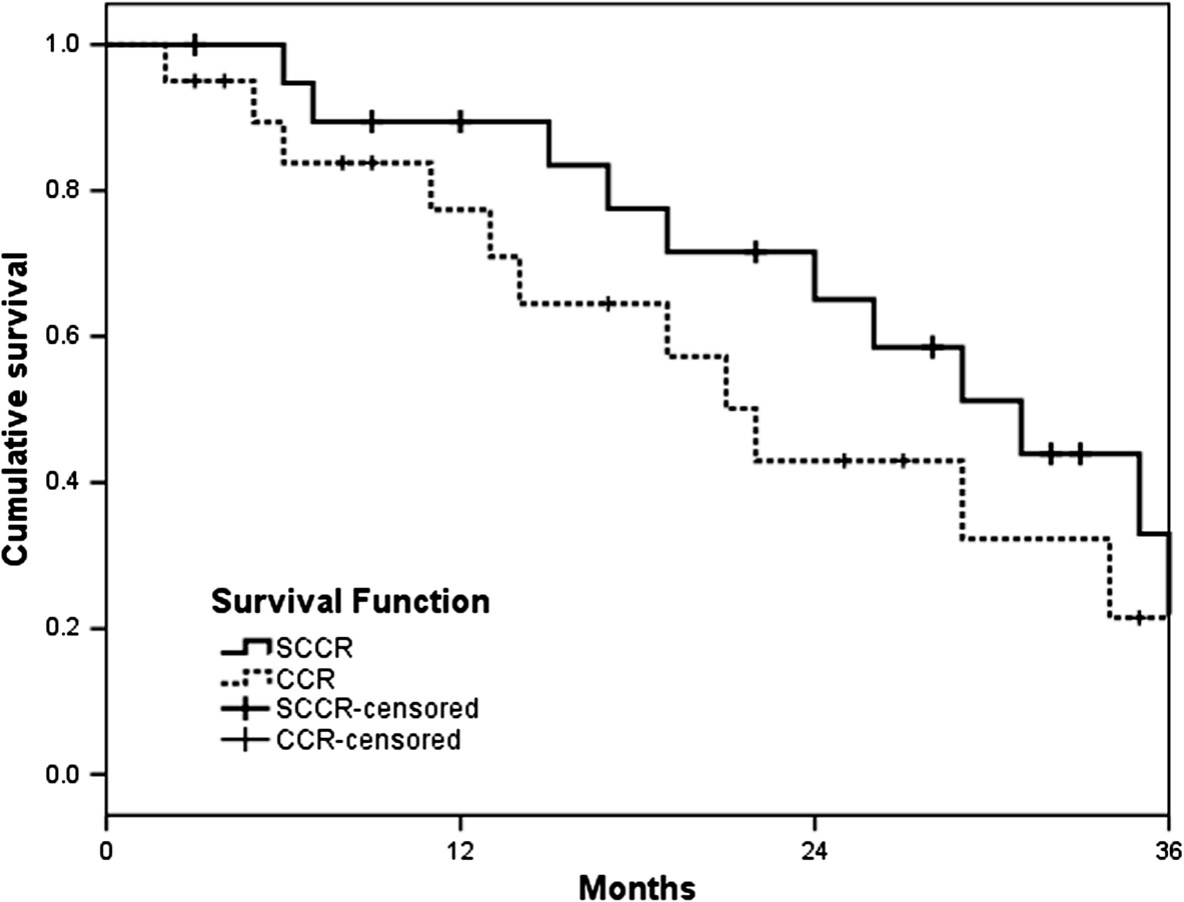
**Progression-free survival.**

The overall response rate as determined by the RECIST criteria was 59.3% for SCCR and 52.4% for CCR; the difference was statistically significant (*P* < 0.05; Table 2). The median OS was 33 months (range, 4–41 months) and 24 months (range, 2–37 months) for the SCCR and CCR groups, respectively (*P* = 0.048; Figure 2). In addition, the median PFS was 31 months and 20 months for the SCCR (range, 5–39 months) and CCR groups (range, 2–37 months), respectively (*P* = 0.037; Figure 3).

**Table 2:** **Summary of adverse events**

**Adverse events**	**SCCR (n = 20)**	**CCR (n = 20)**
	**G3/4 (≥G3) (%)**	**G3/4 (≥G3) (%)**
Haematolgic		
Leukopenia	4/1 (25.0%)	3/1 (20.0%)
Thrombocytopenia	4/0 (20.0%)	3/1 (20.0%)
Neutropenia	4/0 (20.0%)	3/0 (15.0%)
Febrile neutropenia	3/0 (15.0%)	3/0 (15.0%)
Anaemia	3/0 (15.0%)	2/0 (10.0%)
Non-haematolgic		
Anorexia	3/0 (15.0%)	2/0 (10.0%)
Nausea	1/1 (10.0%)	2/0 (10.0%)
Constipation	2/0 (10.0%)	2/0 (10.0%)
Oesophagitis	2/0 (10.0%)	1/0 (5.0%)
Fatigue	1/0 (5.0%)	2/0 (10.0%)
ALT, AST	1/0 (5.0%)	0/0 (0%)
Pneumonitis	1/0 (5.0%)	0/0 (0%)
Diarrhoea	0/0 (0%)	1/0 (5.0%)

All of the AEs that occurred in each group are listed in Table 2. The major hematological toxicities were leucopenia (25.0% in the SCCR group and 20.0% in the CCR group), thrombocytopenia (20.0% in both the SCCR and CCR groups), neutropenia (20.0% in the SCCR group and 15.0% in the CCR group), febrile neutropenia (15.0% in both the SCCR and CCR groups), and anemia (15.0% in the SCCR group and 10.0% in the CCR group). The most common grade 3 or 4 non-hematological toxicities were anorexia (15.0% in the SCCR group and 10.0% in the CCR group), nausea (10.0% in both the SCCR and CCR groups), constipation (10.0% in both the SCCR and CCR groups), and esophagitis (10.0% in the SCCR group and 5.0% in the CCR group). There were no treatment-related deaths in either group.

## Discussion

This is the first pilot randomized controlled study to investigate the use of the oral fluoropyrimidine agent S-1 as a consolidation drug in chemoradiotherapy for stage III NSCLC. Our data indicated reasonable survival with a median survival time of 21.8 months and a three-year survival rate of 34.0%. In addition, tumor response was demonstrated to be 61.5%. However, less than half of the patients completed this regimen (47.6%) and it is unlikely that this treatment is feasible.

Previous studies showed that chemoradiotherapy with cisplatin resulted in high response and survival rates in patients with stage III NSCLC. A phase I trial reported a median survival time of 30.4 months with a three-year survival rate of 50% in 18 patients [12], and a retrospective study showed a median survival time of 21 months and a three-year survival rate of 33% in 73 patients with a median of 2 chemotherapy cycles (mean, 2.4 cycles; range, 1–3 cycles) [13]. A multi-institutional phase II trial of S-1 after concurrent chemoradiotherapy with cisplatin and vinorelbine for locally advanced NSCLC revealed a response rate of 61.5%, a median PFS of 10.2 months (95% confidence interval [CI], 8.6–13.7 months), and a median survival time of 21.8 months (95% CI, 15.6–27.6 months). In addition, the 1- and 3-year survival rates were 73.9% and 34.0%, respectively [14].

SCCR also had a favorable safety profile in this study. The overall frequency of AEs was similar in both groups and most side effects were not severe. The frequencies of both drug-related AEs and severe AEs were higher in the SCCR group than in the CCR group.

Our study has several strengths. First, the trial was randomized thereby reducing selection bias. Second, although there was no consensus regarding the appropriate dose of SCCR for patients with locally advanced NSCLC, the dose used in our study was within the therapeutic range. Further studies with a larger sample size and longer duration of SCCR treatment are needed to further confirm the results of this study.

In conclusion, the results of this study demonstrate promising efficacy and a very acceptable toxicity profile for SCCR in patients with locally advanced NSCLC. Despite the short follow-up period, these encouraging clinical and pathological results warrant further investigation.
